# LMA® protector™ in patients undergoing laparoscopic surgeries: a multicenter prospective observational study

**DOI:** 10.1186/s12871-021-01535-y

**Published:** 2021-12-20

**Authors:** Yanhong Liu, Yuxiang Song, Miaomiao Wang, Meihua Yang, Hao Shen, Zhen Wang, Liyong Chen, Jianjun Yang, Shengkai Gong, Yonghao Yu, Zhao Shi, Wei Zhang, Xuli Zou, Xude Sun, Yuan Wang, Qiang Fu, Jiangbei Cao, Weidong Mi

**Affiliations:** 1 Department of Anesthesiology, The First Medical Center of Chinese PLA General Hospital, Beijing, China; 2 Medical school of Chinese PLA, Beijing, China; 3 Department of Anesthesiology, Daping Hospital, Army Medical University, Chongqing, China; 4 Department of Anesthesiology and Perioperative Medicine, The First Affiliated Hospital of Zhengzhou University, Zhengzhou, China; 5 Department of Anesthesiology, Tianjin Medical University General Hospital, Tianjin, China; 6 Tianjin Research Institute of Anesthesiology, Tianjin, China; 7 Department of Anesthesiology and Perioperative Medicine, Henan Provincial People’s Hospital, Zhengzhou, China; 8 Department of Anesthesiology, The Second Affiliated Hospital of Air Force Medical University, Xi’an, China

**Keywords:** Airway management, Laparoscopy, Laryngeal masks, Supraglottic airway device

## Abstract

**Background:**

Laryngeal masks airway (LMA) has been increasingly used in surgical patients. However, the use of LMA in laparoscopic surgeries remains controversial. The major concerns include the potential risk of esophageal regurgitation, aspiration, and difficulties to achieve effective ventilation. The aim of this study was to evaluate the safety and effectiveness of the LMA® Protector™ in patients undergoing laparoscopic surgery.

**Methods:**

Patients aged 18 to 70 years, scheduled for laparoscopic surgeries were included. The insertion time, successful insertion rate, and oropharyngeal leak pressure were measured. Airway complications and airway manipulations during the procedure were documented. Effective ventilation rate was calculated. Visible bloodstains and reflux content in the drainage channel were documented after the removal of LMA® Protector™.

**Results:**

Three hundred patients were enrolled. The insertion of LMA® Protector™ failed in seven patients resulting with a successful insertion rate of 97.7%. During the maintenance of anesthesia, airway manipulation was required in 19 patients (19/293, 6.48%), in three of whom the LMA was replaced with endotracheal intubation resulting with an effective ventilation rate of 96.7% (290/300). The oropharyngeal leak pressure was 30.18 ± 5.88 cmH_2_O. Seventy-five patients (25.86%) reported mild sore throat on the first day after surgery. Bloodstains on study devices were noticed in 58 patients (20%). Seventy-five patients (25.86%) reported mild sore throat on the first day after surgery. Gastric reflux was noticed in the drainage tube in 5 patients (1.72%) with no signs of aspiration in any of those patients.

**Conclusions:**

The LMA® Protector™ was shown to be safe and effective in patients undergoing laparoscopic surgeries. Although minor complications that require no further treatment, no clinically diagnosed aspiration was noticed in our study. Gastric reflux was noticed in the drainage tube in five patients undergoing laparoscopic gynecology surgery. Further research is needed to verify whether LMA® Protector™ is suitable for procedures in Trendelenburg position or other situations that a high risk of gastroesophageal reflux exists.

**Trial registration:**

The trial was registered at the Chinese Clinical Trial Registry (ChiCTR1800018300, date of registration: September 2018).

## Introduction

Laparoscopic surgery is now being used for more varieties of surgery than ever before due to the advantages that it offers over conventional open surgery. General anesthesia with endotracheal intubation is used as a common practice in this kind of surgery. As an alternative to endotracheal intubation, the laryngeal mask airway (LMA) has also been proved to be safe and may have several advantages in these procedures, including improved hemodynamic and respiratory stability, less restricted mucociliary clearance, and a reduced need for anesthetics [1–7]. However, there remain some concerns with the use of LMA in laparoscopic surgery. The major concerns include the potential risk of gastroesophageal regurgitation, aspiration, and difficulties to achieve effective ventilation due to the influence of artificial pneumoperitoneum and postural changes on airway pressure and pulmonary compliance [1, 2]. Therefore, many anesthesiologists advocate endotracheal intubation and mechanical ventilation for this kind of procedures despite evidences provided by previous studies.

It has been shown that the performance of a laryngeal mask can be affected by different factors, including types of materials, sizes of esophageal drainage tubes, intracuff pressure, and shapes of the cuff [1, 6, 8–10]. Numerous attempts have been made to improve the sealing pressure and drainage of reflux, and therefore, to reduce the risk of air leak and aspiration of LMAs [11]. LMA® Protector™ (Teleflex Medical Europe, Westmeath, Ireland) was first used in clinical in 2015. It is made of medical-grade silicone with two large-volume gastric drainage channels and integrated with a cuff pressure indicator, the Cuff Pilot™ [12]. Previous studies have assessed the clinical performance of LMA® Protector™ [12, 13]. This second-generation LMA has enabled the application of higher respiratory pressure, possible drainage of regurgitated material and the introduction of a gastric tube via integrated gastric access [8, 12, 14, 15].

Recent studies also showed that LMA® Protector™ provides effective and efficient pulmonary ventilation in laparoscopic surgeries, promising as an alternative to endotracheal intubation in such procedures. However, due to the low incidence, adverse events that correlated with the use of LMA® Protector™, such as failed ventilation, esophageal regurgitation, pulmonary aspiration, postoperative sore throat, and other airway complications, were not intensively monitored in previous studies. To evaluate the prevalence of these complications, a large scale research was usually required. Therefore, in this multicenter study, we further investigated the effectiveness and safety of LMA® Protector™ in laparoscopic surgeries in a large sample and intended to provide evidence for LMA to be used in laparoscopic surgery [3, 16].

## Materials and methods

### Study design

This study was conducted in six hospitals in China (The First Medical Center of Chinese PLA General Hospital, Henan Provincial People’s Hospital, The First Affiliated Hospital of Zhengzhou University, Tianjin Medical University General Hospital, The Second Affiliated Hospital of Air Force Medical University, Daping Hospital) from October 01, 2018, to October 31, 2019. The study was registered at the Chinese Clinical Trial Registry on September 10, 2018 (ChiCTR1800018300, Weidong Mi as the principal investigator). The study adhered to the STROBE Statement and was conducted in accordance with the Declaration of Helsinki. Written informed consent was obtained from each participant before enrollment.

### Ethics

This study was approved by the ethics committee of Chinese PLA General Hospital(S2017–034-02), Daping Hospital of Army Medical University (2018–45), the First Affiliated Hospital of Zhengzhou University (SS-2019-038), Tianjin Medical University General Hospital (record), Henan Provincial People’s Hospital (2018–46), the Second Affiliated Hospital of Air Force Medical University (201812–09).

### Study population

In this study, we included adult patients who were American Society of Anesthesiology (ASA) class 1 and 2, 18 to 70 years old, and scheduled for laparoscopic surgeries with an expected duration of surgery less than 4 h and expected blood loss less than 300 ml. Exclusion criteria consisted of BMI > 30 kg/m^2^, patient refusal, a suspected difficult airway, increased risk of aspiration (hiatus hernia, gastroesophageal reflux disease, non-fasting status), recent upper respiratory tract infection, inability to comply with study requirements including follow-up. All the anesthetists who participated in this study were experienced in the use of LMA with more than 200 cases of LMA insertions and were trained in the use of LMA® Protector™ (the study device) and study protocol before the beginning of the study.

### Study procedures

All patients received standard monitoring, including electrocardiogram, non-invasive blood pressure, and oxyhemoglobin saturation in the operation room. After pre-oxygenation with a facemask for 3 min, anesthesia was induced with sufentanil 0.2–0.4 μg/kg or fentanyl 1-3 μg/kg, propofol 1-2 mg/kg oretomidate 0.2–0.3 mg/kg, and rocuronium 0.6–0.9 mg/kg or cisatracurium 0.2–0.3 mg/kg. LMA® Protector™ insertion was attempted in all patients. The choice of LMA® Protector™ size was based on the following principles: weight ≤ 50 kg for size 3; weight between 51 and 70 kg for size 4; weight>70 kg for size 5. After deflation and lubrication of the cuff, the LMA® Protector™ was inserted with the distal tip kept in the midline until resistance was felt. Then the cuff was inflated to maintain the intracuff pressure at 40 to 60 cmH_2_O according to the Cuff Pilot™, the integrated cuff pressure indicator [17]. The anesthesia apparatus was connected to the LMA® Protector™ for mechanical ventilation. Chest wall movement and capnography were used to confirm the successful placement of LMA. Further extension of the head and chin lift performed by the assistant was permitted during the insertion of the study device.

The insertion time was measured from the moment the anesthetist picked up the LMA® Protector™ until the appearance of the first capnography trace by an assistant. Oropharyngeal leak pressure (OLP) was measured by setting the adjustable pressure limiting valve to 40 cmH_2_O at a fixed gas flow of 3 L/min and noting the steady-state airway pressure on the monitor [10, 18]. The suprasternal notch test was performed as Eckardt described, [19] and a lubricated 14F gastric tube was then inserted through the female drainage port. Failed insertion of LMA® Protector™ was defined by any of the following criteria: [1] failed passage into the pharynx [2]; malposition (air leaks or end-tidal capnography could not be obtained); and [3] ineffective ventilation (maximum expired tidal volume < 8 ml/kg, end-tidal carbon dioxide > 45 mmHg, or pulse oxygen saturation < 92% if correctly positioned) [13]. We allowed a maximum of three attempts with the allocated device. The easiness of insertion was graded as easy, mild difficulty with resistance, moderate difficulty with resistance, and severe difficulty with resistance.

Anesthesia was maintained with intravenous or intravenous-inhalation combined anesthesia to keep bispectral index (BIS) between 40 and 60. Supplemental doses of neuromuscular blockade (rocuronium 0.1–0.2 mg/kg or cisatracurium 0.02–0.04 mg/kg) was administered intermediately during the operation. The timing of muscle relaxants during the operation was guided by clinical criteria according to the pharmacokinetics of muscle relaxants, the pathophysiological characteristics of patients, and the demand for surgical relaxation. The total dosage of neuromuscular blockade used during the surgery was recorded. Volume control ventilation was set at an inspired tidal volume of 6–8 mL/kg, a respiratory rate of 12/min, and an inspiratory/expiratory ratio of 1:2 to maintain the end-tidal CO_2_(EtCO_2_) at around 35–45 mmHg and no positive-end-expiratory pressure was used. Pneumoperitoneum was established by insufflation of carbon dioxide to a pressure of 14 mmHg. During maintenance of anesthesia, airway complications (intermittent obstruction, complete obstruction, airway leak) and airway manipulations during the procedure (position corrections, additional cuff inflation or deflation, device reinsertion) were documented. If effective ventilation could not be achieved after airway manipulations, i.e., maximum expired tidal volume < 8 ml/kg, end-tidal carbon dioxide > 45 mmHg, or pulse oxygen saturation < 92%, the LMA® Protector™ would be replaced with endotracheal intubation. Effective ventilation rate was calculated as the proportion of patients who were effectively ventilated with LMA® Protector™ throughout the procedure in all participants.

On emergence from anesthesia, the LMA® Protector™ was removed when the patient was able to breathe spontaneously and follow verbal instructions. All patients were monitored for at least 30 min in the recovery room before returning to the ward. Visible bloodstains and reflux content in the drainage channel were documented after the removal of LMA® Protector™. The operator’s satisfaction score was rated on a scale of 0 (worst) to 10 (best) by the anesthetists. On the first day after surgery, participants were interviewed to ascertain if they had the following complaints: sore throat, dysphagia, and hoarseness of voice. Vigilance against aspiration was kept especially when the patient presented with symptoms such as coughing, difficulty breathing, choking, or wheezing. Further evaluation would be taken if aspiration was suspected. Adverse events should be reported if the patient was diagnosed as aspiration during hospitalization or re-administered as aspiration pneumonia in 1 month after surgery.

### Statistical analysis

The sample size was calculated based on the primary outcome of effective ventilation rate. An estimated minimum sample size of 243 patients would be required to show an effective ventilation rate of 95% with a single-sided type I error of 0.05 and power of 0.8 [20]. As the sample dropout rate was estimated to be 20%, the final determined sample size was 300.

The Kolmogorov-Smirnov test was performed to evaluate the normality of continuous data. Mean and the standard deviation was used to describe symmetrically distributed continuous data. Asymmetrically distributed continuous data were presented as median [interquartile range]. Percentages were used to describe categorical data. Continuous data were analyzed with Student’s *t* test or the Mann-Whitney *U* test. The chi-square test or Fisher’s exact test was used for categorical data, as appropriate. All the data were analyzed using SPSS (Version 20, IBM Corp., Chicago, IL, USA).

## Results

**Fig. 1:**
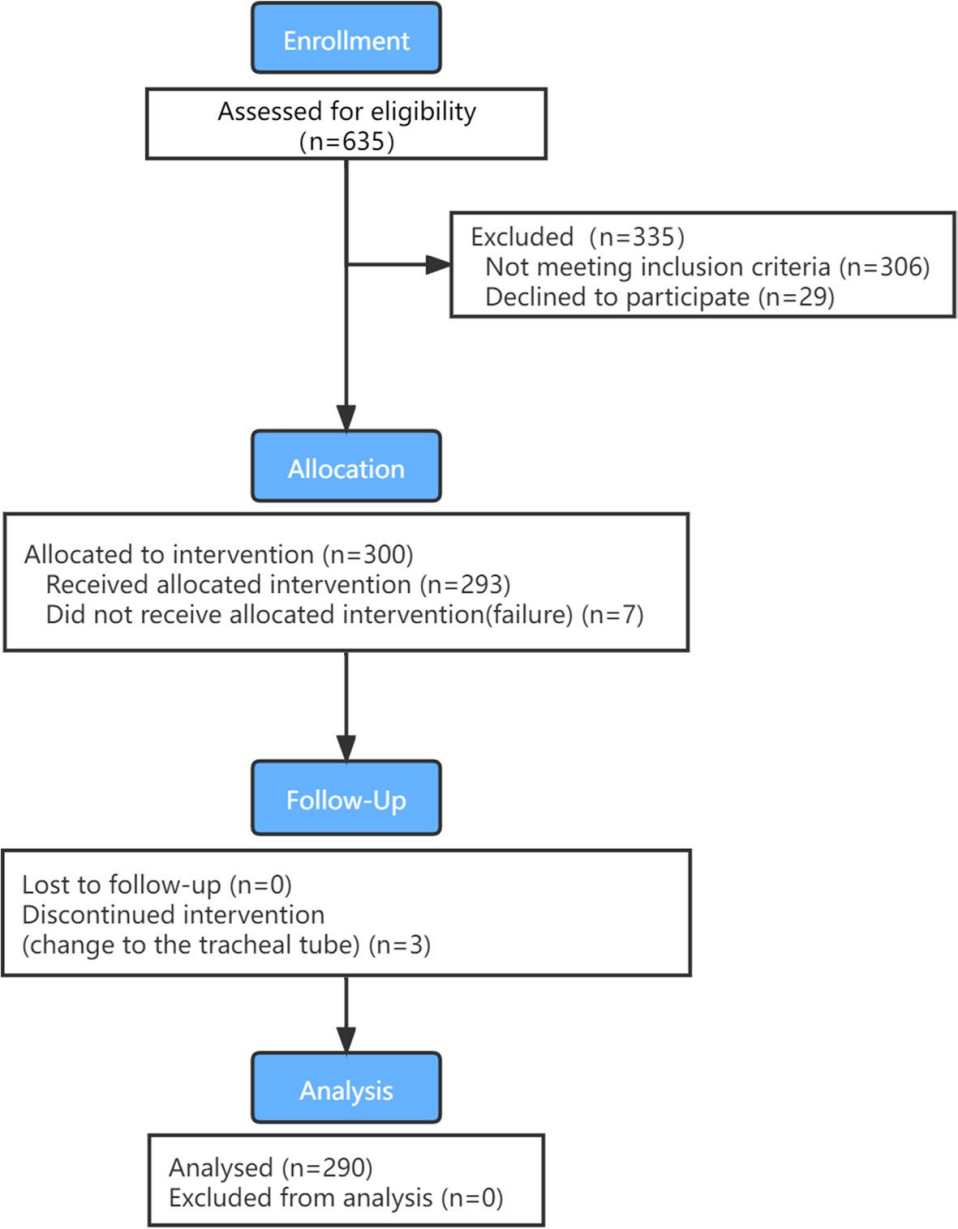
Patient enrolment and flow chart

Figure 1 shows patient enrollment and flow chart. Three hundred patients were enrolled. The characteristics of these patients are presented in Table 1. The insertion of LMA® Protector™ failed after three attempts in seven patients (7/300, 2.41%). During the maintenance of anesthesia, airway manipulation was required in 19 patients (19/293, 6.48%), in three of whom the LMA was replaced with endotracheal intubation. The effective ventilation rate of LMA® Protector™ was 96.7% (290/300). Comparison of demographic parameters between successful and failed cases showed no difference (Table 1).

**Table 1:** Characteristics of Subjects recruited

Parameters	Successful cases (***n*** = 290)	Failed cases (***n*** = 10)	***P*** value
**Age (years)**	43.78 ± 12.74	48.10 ± 11.93	0.2917
**Male/Female,** ***n***	72/218	1/9	0.4603
**Body weight (kg)**	62.39 ± 10.76	58.35 ± 8.11	0.2409
**Body mass index (kg m**^**−2**^**)**	23.46 ± 3.32	22.75 ± 3.41	0.5070
**ASA status,** ***n*** **(%)**			0.8467
**Class 1**	136 (46.9%)	5 (50%)	
**Class 2**	154 (53.1%)	5 (50%)	
**Mallampati Classification,** ***n*(%)**			0.3896
**1**	135 (46.55%)	3 (30%)	
**2**	142 (48.97%)	7 (70%)	
**3**	13 (4.48%)	0	
**Type of Surgery, n (%)**			0.7955
**Gallbladder surgery**	94 (32.41%)	3 (30%)	
**Gynecology**	147 (50.69%)	6 (60%)	
**General surgery**	49 (16.9%)	1 (10%)	
**Position of the patients,** ***n*** **(%)**			0.9632
**Supine position**	23 (7.93%)	1 (10%)	
**Reverse-Trendelenburg position**	95 (32.76%)	3 (30%)	
**Trendelenburg position**	172 (59.31%)	6 (60%)	
**Duration of Surgery (min)**	81.14 ± 41.30	–	
**Total dosage of neuromuscular blockade (mg)**			0.8840
**Rocuronium**	48.62 ± 13.26	48.33 ± 16.02	
**Cisatracurium**	14.82 ± 5.17	13.5 ± 1.66	

Data presented as mean (SD) or absolute numbers (%). *ASA* American Society of Anesthesiologists

The performance of the LMA® Protector™ is shown in Table 2. The recommended size of the LMA® Protector™ was suitable in 285 patients (285/300, 95%). The median insertion time was 29.5 s. The oropharyngeal leak pressure was ≥25 cmH_2_O in 85% of patients. The anesthetists’ satisfaction score of LMA® Protector™ was 8.59 ± 1.02.

**Table 2:** Insertion and ventilation parameters

Parameters	Result
**Successful insertion,** ***n*** **(%)**	293 (97.67%)
1st attempt	251 (85.67%)
2nd attempt	36 (12.29%)
3rd attempt	6 (2.05%)
Failed	7 (2.33%)
**Size,** ***n*** **(%)**	
3	172 (59.31%)
4	113 (38.97%)
5	5 (1.72%)
**Size change,** ***n*** **(%)**	
No	283 (97.59%)
Yes	7 (2.41%)
**Time of insertion (s)**	29.5 [15 to 60]
**Easiness of insertion,** ***n*** **(%)**	
Easy	41 (14.14%)
Mild difficulty with resistance	149 (51.38%)
Moderate difficulty with resistance	97 (33.45%)
Severe difficulty with resistance	3 (1.03%)
**Suprasternal notch test,** ***n*** **(%)**	
Positive	219 (75.52%)
Negative	71 (24.48%)
**Successful gastric tube insertion,** ***n*** **(%)**	
1st attempt	215 (74.14%)
2nd attempt	47 (16.21%)
3rd attempt	21 (7.24%)
Failed	7 (2.41%)
**Oropharyngeal leak pressure (cmH**_**2**_**O)**	30.18 ± 5.88
**Manipulation of the airway,** ***n*** **(%)**	19 (6.48%)
Adjust the position	13 (68.42%)
Additional cuff Inflation/deflation	2 (15.38%)
Device reinsertion	1 (7.69%)
Replacement of device	3 (23.08%)
**Effective ventilation rate,** ***n*** **(%)**	290 (96.67%)
**Operator’s Satisfaction**	8.59 ± 1.02

Data presented as Mean ± SD, median [interquartile range], or absolute numbers/(percentage). Operator’s satisfaction graded on a scale of 0 (worst) to 10 (best) by the anesthetists

Table 3 shows the most frequent complications of LMA® Protector™ observed in this study. Bloodstains on study devices were noticed in 58 patients (20%). Seventy-five patients (25.86%) reported mild sore throat on the first day after surgery. All of them recovered and no further treatment was required. Gastric reflux was noticed in the drainage tube in 5 patients (1.72%) with no signs of aspiration in any of those patients.

**Table 3:** Complications of LMA® Protector™ after surgery

Parameters	Number (percentage)
**Complications in post-anesthesia care unit,** ***n*(%)**	
Bloodstains on device	58 (20%)
Reflux content in drainage	5 (1.72%)
**Complications on POD1,** ***n*** **(%)**	
**Sore throat**	
Mild	75 (25.86%)
Moderate	0
Severe	0

Data presented as absolute numbers/(percentage). POD1: the first day after surgery

## Discussion

In the present study, we found that LMA® Protector™ was an effective supraglottic airway device (SAD) in laparoscopic surgeries. The initial successful insertion rate was 97.7%, which is relatively high in comparison to other SADs from the literature review. During the maintenance of anesthesia, the airway manipulation frequency was as low as 6.48%. The oropharyngeal leak pressure was 30.18 ± 5.88 cmH_2_O and could achieve effective ventilation in 96.7% of patients throughout the procedure.

The mean oropharyngeal leak pressure of LMA® Protector™ in our study was 30 cmH_2_O, which was in keeping with the previous findings on LMA® Protector™ by Zaballos, M., [21] and van Zundert [18]. The oropharyngeal leak pressure of 30 cmH_2_O was better than most of the other LMAs that were commonly used in clinical practice and meet the needs of laparoscopic surgeries [22, 23]. In our study, once the LMA® Protector™ was placed properly, the frequency of airway manipulation was very low during the maintenance of anesthesia. Adequate ventilation could be achieved with LMA® Protector™ in patients in the Trendelenburg position as well. The advantage of the study device in high oropharyngeal leak pressure and low frequency of airway manipulation may partially be attributed to the silicone material that is used in LMA® Protector™, which makes the device soft and flexible [11]. The integrated Cuff Pilot™ with a cuff pressure indicator that provides easier adjustment of the intracuff pressure may also be beneficial in reducing LMA displacement and air leak [12, 17].

A major concern of using LMAs in laparoscopic surgery is the risk of regurgitation and aspiration [2]. The incidence of regurgitation varies from 0.2 to 16.7% in previous reports with different SADs [9, 24, 25]. In this study, gastric reflux was noticed in five patients (1.72%) in the drainage tube of the device at the end of surgery. All of the five patients were undergoing laparoscopic gynecology surgery in the Trendelenburg position. We believe that the patient’s postural status and pneumoperitoneum during surgery were major causes of regurgitation, although previous studies have proved that LMA could be used safely in laparoscopic gynecology surgery in a head-down position [1, 10]. None of these patients experienced any signs of aspiration or pulmonary complications. It is believed that the large volume drainage channels built in LMA® Protector™ had possibly made the reflux easier to drain out and therefore reduced the risk of aspiration [26]. Further research is needed to verify this point of view. However, our findings suggested that attention should be given to gastroesophageal regurgitation especially under head-down positions and adequate suction should be conducted before removal of SADs. The incidence of sore throat (25.86%) observed in our study on the first day after surgery was relatively higher in comparison with 6% in Zaballos, M.’s study [21] and 23.1% in Sng’s study [13]. However, all 75 patients experienced mild symptoms and recovered without any further intervention.

This study showed that the median insertion time was 29.5 s, which seems to be longer than what was reported in previous studies (4.8 s to 19 s) [12, 13]. The reasons for this discrepancy may include the following: First, a different definition of insertion time was used. In our study, the insertion time was defined as from picking up the LMA® Protector™ until the first capnography trace appeared, which included insertion, inflation, and connection of the ventilation machine, other than inserting alone [12]. Second, LMA® Protector™ was designed according to the anatomical characteristics of Caucasians. At the same time, the silicone material and double drainage channel integrated in LMA® Protector™ had made it softer and wider. In our survey of the operators, the difficulty of insertion was more than moderate in 34.81% of cases. A small amount of blood was noticed on the device in 58 (20%) patients after the removal of LMA® Protector™. These indicated that the difference in airway anatomy between Asians and Caucasians might have affected the difficulty of insertion. Third, although all the operators were experienced with LMA and were trained before the beginning of the study, there was still a learning curve in the use of LMA® Protector™. As the number of cases increased, the insertion time began to shorten gradually.

There were several limitations in our study. First, the position of LMA® Protector™ was not determined by fiberoptic bronchoscopy, which may provide a more objective diagnosis of malposition than the clinical signs that were used in our study. Second, we used clinical criteria to guide the use of neuromuscular blockade rather than the neuromuscular block monitoring, which may lead to different degrees of muscle relaxation between patients. Third, we did not compare the LMA® Protector™ with other SADs or endotracheal intubation in this study. However, for our intention to evaluate the efficacy and safety of LMA protector in laparoscopic surgeries, we have well controlled the quality of our study and have make sure that all investigators were trained prior to the study. The oropharyngeal leak pressure in our study was shown to be comparable to other LMAs reported in previously published articles [22, 23]. We believe that our results, including the prevalence of failed ventilation, airway manipulation, and postoperative complications, are of great help to clinicians in choosing airway management tools for patients undergoing laparoscopic surgeries.

In conclusion, LMA® Protector™ was shown to be safe and effective in patients undergoing laparoscopic surgeries. The high sealing pressure provided by the study device guarantees effective mechanical ventilation during pneumoperitoneum. No clinically diagnosed aspiration was noticed in our study except for minor complications, such as sore throat and bloodstaining on devices. Gastric reflux was noticed in the drainage tube in five patients undergoing laparoscopic gynecology surgery. Further research is needed to verify whether LMA® Protector™ is suitable for procedures in the Trendelenburg position or other situations that a high risk of gastroesophageal reflux exists.

## Conclusions

The LMA® Protector™ was shown to be safe and effective in patients undergoing laparoscopic surgeries. Although minor complications that require no further treatment, such as sore throat were reported, no clinically diagnosed aspiration was noticed in our study.

## Data Availability

The datasets and materials related to the current study are available from the corresponding author upon reasonable request.

## Abbreviations

LMA: Laryngeal masks airway
SAD: Supraglottic airway device
OLP: Oropharyngeal leak pressure
ASA: American Society of Anesthesiologists
